# Printable and Stable Microlens‐Type Perovskite Quantum Dot Color‐Converting Resin Through Rheology Control for Efficient and Reliable Micropatterning for Full‐Color Displays

**DOI:** 10.1002/smll.74347

**Published:** 2026-06-26

**Authors:** Yongmin Shin, Jiho Joo, Byung Jo Um, Jung Ho Shin, Chanmi Lee, Somin Park, Junho Jang, Byeong‐Soo Bae

**Affiliations:** ^1^ Wearable Platform Materials Technology Center (WMC) Department of Materials Science and Engineering Korea Advanced Institute of Science and Technology (KAIST) Daejeon Republic of Korea; ^2^ Superintelligence Creative Research Laboratory Electronics and Telecommunications Research Institute (ETRI) Daejeon Republic of Korea; ^3^ Insulation Materials Research Center Electrical Materials Research Division Korea Electrotechnology Research Institute (KERI) Changwon‐si Gyeongsangnam‐do Republic of Korea

**Keywords:** color‐conversion, micro‐LED display, perovskite quantum dot, siloxane hybrid, stability, thixotropic resin

## Abstract

Perovskite quantum dots (PQDs) are promising color‐converting materials for full‐color micro‐light‐emitting diode (micro‐LED) displays; however, PQDs have intrinsic poor stability which hinders practical applications. In addition, micro‐LEDs that incorporate color‐converting layers with reliable operation remain limited. Herein, we report a highly‐stable thixotropic PQD‐based color converting resin (thixo‐PQD resin) by precise control of rheological properties and robust matrix encapsulation. Fumed silica nanoparticles (FSNs) are incorporated in thixo‐PQD resin that serve as thixotropic and scattering agents, thereby forming microlens thixo‐PQD arrays (ML thixo‐PQD arrays). The resulting ML thixo‐PQD arrays retain high photoluminescent quantum yield (PLQY ∼ 78%) in ambient air, under 85°C/85% RH, and under continuous blue‐light irradiation for > 2400 h. This stability of the ML thixo‐PQD array arises from synergistic effects of (i) effective physical blocking of water and oxygen by the FSN network and encapsulating matrix and (ii) chemical interactions between the PQD surface ligands and the encapsulating matrix. To confirm the feasibility as a color‐converting layer on micro‐LED, we print ML thixo‐PQD arrays directly onto blue‐emitting micro‐LED backplanes; the devices achieve a 59.2% increase in luminous flux and reliability under 85°C/85% RH for > 60 d. These thixo‐PQDs have potential application in high‐resolution, full‐color micro‐LED displays.

## Graphical abstract

A thixotropic perovskite quantum dot color‐converting resin (thixo‐PQD resin) is developed by rheological control and robust matrix encapsulation, enabling uniform micro‐dispensing of microlens (ML) thixo‐PQD array. The resulting ML thixo‐PQD array shows improved color‐conversion relative to flat PQD films and achieves long‐term environmental stability. Incorporating thixo‐PQD resin material achieved a color‐converted micro‐LED that has efficiency and reliability.

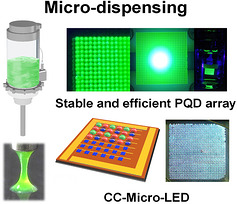

## 1 Introduction

Micro‐LED, with very high resolution under 100 µm pixel size, has been extensively researched for high‐resolution displays because of high contrast ratio, low operating power, and high luminous efficiency [1, 2]. These characteristics provide superior color fidelity and potentially high luminance, and therefore make micro‐LEDs ideal for advanced display applications such as augmented reality (AR), virtual reality (VR), and wearable devices [3, 4, 5]. Despite these advantages, the transfer of individual micro‐LED chips is not sufficiently precise, and the epitaxial growth of materials (e.g., GaN or InGaN) is not sufficiently controlled; these technical problems significantly impede the manufacturing of micro‐LEDs [6]. In particular, red‐emitting micro‐LEDs suffer from severe non‐radiative recombination at etched sidewalls [7], and therefore are less reliable than blue‐emitting micro‐LEDs. To overcome these limitations, color‐converted micro‐LED (CC‐micro‐LED) displays have been developed; they use a blue‐emitting micro‐LED as the excitation source, and green and red‐emitting components are generated by optical down‐conversion in a color‐conversion layer (CCL) [3, 8].

To fabricate CCLs that incorporate high‐resolution quantum dots (QDs) or perovskite nanocrystals (PQDs), several approaches have been investigated; examples include photolithography [9, 10, 11], inkjet printing [12, 13], microfluidic printing [14, 15], and transfer printing [16, 17]. However, these methods have demerits, including the need for flip‐chip bonding or precise alignment processes that can damage the micro‐LED chip [18, 19]. The inkjet printing of low‐viscosity PQD inks is problematic because solvent evaporation during the printing process can reduce the uniformity of both pixel thickness and surface curvature [20].

Printable inks that incorporate PQDs should have high stability while maintaining patterning resolution, and the inks must also be compatible with printing on micro‐LED backplanes [3, 21]. In practice, dilute or weakly‐passivated inks may yield high‐resolution pixels that degrade rapidly, so resolution and chemical stability are often in a trade‐off relation [22]. Consequently, chemical compatibility between PQD surface ligands and the encapsulating matrix becomes critical, providing robust protection of PQD from water or oxygen molecules under humid environments. Direct ink writing (DIW) allows printing of high‐viscosity PQD inks to achieve precisely controlled structures while minimizing material waste [23, 24]. In particular, micro‐dispensing of highly viscous, thixotropic resins enables formation of microlens (ML) arrays, in which internal reflections are reduced, and light scattering is increased; these effects increase light‐extraction efficiency and color‐conversion efficiency [25, 26, 27]. This method improves the optical characteristics of the CCLs while ensuring compatibility with micro‐LED backplanes [28, 29].

This work presents development of a high‐viscosity thixotropic PQD/siloxane resin (thixo‐PQD resin) that has excellent color‐converting characteristics, and has strong potential for use in efficient and reliable micro‐LEDs or other advanced backlight units. Formulation of the thixo‐PQD resin entails two steps: (i) in situ sol–gel condensation that yields a dense siloxane hybrid matrix that protects PQDs, and (ii) blending fumed‐silica nanoparticles (FSNs) into the resin to impart non‐Newtonian rheology such as thixotropy and shape retention. A micro‐dispensing process is used to uniformly print this thixo‐PQD resin to yield microlens‐shaped PQD array films (ML thixo‐PQD array). The color‐conversion efficiency of the fabricated ML thixo‐PQD array is improved due to scattering effects and the three‐dimensional (3D) ML architecture. Moreover, the ML thixo‐PQD array retained their optical properties under water immersion, under 85°C/85% RH, and under continuous blue light irradiation, demonstrating robust environmental stability. This significant stability increase is attributed to defect passivation at PQD surface and to effective protection by the siloxane hybrid matrix and FSN network. To confirm the feasibility of the thixo‐PQD resin, we demonstrate a full‐color color‐converted micro‐LED (CC‐micro‐LED) by printing a green‐emitting ML thixo‐PQD array, a red‐emitting ML thixo‐RQD array, and a transparent ML thixo‐QD‐free array (for blue pixels) directly onto a blue‐emitting micro‐LED backplane. The fabricated CC‐micro‐LED has 59.2% improved luminance flux with exceptionally high reliability under 85°C/85% RH for 60 d; this result demonstrates that the thixo‐PQD resin is an efficient color‐converter. These thixo‐PQD color‐converting resins can be used to efficiently and reliably print color‐converted micro‐LED displays.

## 2 Results and Discussion

### 2.1 Synthesis of Thixo‐PQD Resin

**FIGURE 1:**
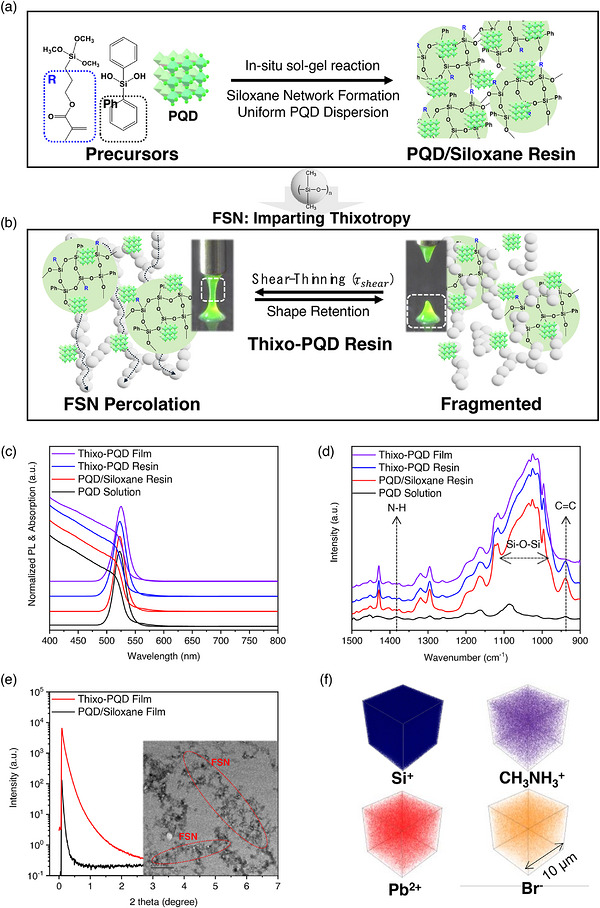
(a) Schematic illustration of PQD/siloxane resin synthesized by in situ sol–gel reaction. (b) FSN percolation network of thixo‐PQD resin (left) and FSN fragmentation of thixo‐PQD resin (right). Insets: micro‐dispensing images when shear stress τ_*shear*_ is applied and not applied. (c) PL and absorption spectra of PQD solution (black), PQD/siloxane resin (red), thixo‐PQD resin (blue), and thixo‐PQD film. (d) FT‐IR spectra of PQD solution (black), PQD/siloxane resin (red), and thixo‐PQD resin (blue). (e) SAXS patterns of thixo‐PQD film (red) and PQD/siloxane film (black). Inset: TEM image of thixo‐PQD film indicating FSN percolation and PQD dispersion (scale bar: 200 nm). (f) TOF‐SIMS 3D elemental mapping of Si^+^ (blue), CN^−^(green), Pb^2+^(red), and Br^−^ (yellow) in 1000 µm^3^ cube.

At first, MAPbBr_3_ PQDs passivated by methacrylate‐functionalized siloxane‐derived ligand were synthesized using a previously reported method [30]. The resulting colloidal PQDs had an emission wavelength *λ* ∼ 523 nm with a narrow bandwidth of ∼ 25 nm, and a PLQY of ∼ 80% (Table S1). Thus, the optical characteristics of the PQD colloidal solution are compatible with subsequent thixo‐PQD formulation and micro‐dispensing. The PQD/siloxane resin was then prepared (Figure 1a) by an in situ sol–gel reaction between the PQD solution and two silane precursors (3‐methacryloxypropyl) trimethoxysilane (MPTS) and diphenylsilanediol (DPSD). During this process, the PQDs became molecularly dispersed within the growing siloxane hybrid network; we attribute this result to hydrophobic interactions between the PQD surface ligands and the phenyl groups of DPSD [31, 32].

To impart the shear‐thinning behavior and shape fidelity that are required for micro‐dispensing, fumed silica nanoparticles (FSNs) were incorporated into the PQD/siloxane resin, to yield a thixotropic PQD resin (thixo‐PQD resin) (Figure 1b). The thixo‐PQD resin was micro‐dispensed through a fine needle (inner diameter = 150 µm) by using an iterative gap‐adjustment and extrusion cycle to form a uniform ML thixo‐PQD array. Under applied shear stress, the FSN network within the thixo‐PQD resin forms a percolated structure along the shear direction, and thereby enables uniform dispensing. When the shear stress is removed, the FSN network fragments into smaller clusters; this process allows the resin to retain its shape. The printed ML thixo‐PQD arrays were UV‐cured to induce free‐radical polymerization between MPTS and methacrylate groups on the PQD ligands.

The PL spectra (Figure 1c) of the PQD solution, PQD/siloxane resin, and thixo‐PQD resin have nearly identical emission peak positions and spectral bandwidths, with only a slight peak shift relative to the original PQD solution (Table S1). This near‐invariance of the emission profiles indicates that neither the in situ sol–gel condensation nor the subsequent UV‐curing induces ligand dissociation or halide degradation.

Fourier Transform‐Infrared (FT‐IR) spectra (Figure 1d) provide further information about the chemical environment and network structure surrounding the PQDs. The PQD solution shows characteristic ligand binding, including amine‐associated features at ∼1380 cm^−1^ [30]. After formation of the PQD/siloxane and thixo‐PQD resins, the spectra show strong Si‐O‐Si stretching bands at 1000–1130 cm^−^^1^, which indicate condensation of the siloxane network. Incorporation of FSN intensifies these bands; this change is evidence that the silica surface contributes to the siloxane hybrid framework. Meanwhile, thixo‐PQD resin retains a peak at 940 cm^−1^ (C═C bond at methacrylate groups), whereas the UV‐cured thixo‐PQD film shows this peak was removed. Together with ^2^ ^9^Si‐Nucelar Magnetic Resonance (^2^ ^9^Si‐NMR) analysis (Figure S1), these results are evidence for the formation of a highly condensed siloxane network that mechanically and chemically protects the MAPbBr_3_ PQDs.

The crystalline structure of the MAPbBr_3_ nanocrystals and the mesoscale structure of the FSN network were elucidated by a combination of powder X‐ray Diffraction (XRD), Small‐Angle X‐ray Scattering (SAXS), and Transmission Electron Microscope (TEM) analyses. Powder XRD patterns of the PQD/siloxane and thixo‐PQD films (Figure S2) display MAPbBr_3_ NC diffraction peaks [33], which indicate that the crystalline MAPbBr_3_ phase is preserved within the siloxane hybrid matrix. The SAXS curves (Figure 1e) exhibit characteristic q‐dependent scattering features that can be converted to real‐space correlation lengths (d‐spacings), which indicate a hierarchical microstructure in which PQDs are uniformly dispersed within a percolated FSN network [34, 35]. These results were used to estimate that the characteristic FSN domain size is on the order of 300–500 nm. In contrast, a PQD/siloxane film without FSN did not show hierarchical microstructure. This morphology is corroborated by TEM images (Figure 1e, inset; Figure S3) of the thixo‐PQD film, which reveal an interconnected FSN network with PQDs embedded within it. This continuous percolated FSN network promotes shear‐induced alignment under flow, and thereby facilitates formation of stable filaments during micro‐dispensing through the fine needle. Time‐of‐flight Secondary‐ion‐mass‐spectroscopy (TOF‐SIMS, Figure 1f) and dispersive PL/Raman mapping results (Figure S4) also support uniform dispersion of the PQDs and FSNs in the siloxane hybrid matrix. These results are evidence for the structural and optical homogeneity of the thixo‐PQD resin and thixo‐PQD films.

### 2.2 Rheological Properties of Thixo‐PQD Resin

**FIGURE 2:**
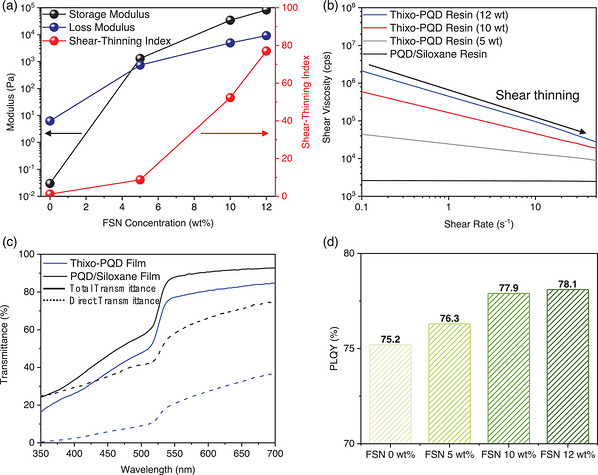
(a) Storage modulus (black), loss modulus (blue), and shear‐thinning index (red) of PQD/siloxane resin and thixo‐PQD resin. (b) Viscosity flow curve of PQD/siloxane resin and thixo‐PQD resin. (c) Transmittance spectra of thixo‐PQD film (blue) and PQD/siloxane film (black). Solid lines: total transmittance spectra; broken lines: direct transmittance spectra. (d) PLQY of PQD/siloxane film and thixo‐PQD film.

To achieve reliable ML formation, the dispensed resin must retain its structure without flowing or collapsing. Rheological properties of thixo‐PQD resin by FSN content were investigated to confirm that the formulation of thixo‐PQD resin is printable by micro‐dispensing (Figure 2a,b and Table 1). The damping factor *ζ* is defined as the ratio of loss modulus G" to storage modulus G' at rest:

(1) $$\zeta =\frac{G^{\prime \prime }}{G^{\prime }}\;$$

*ζ* quantifies the resin's ability to preserve its printed geometry after micro‐dispensing. A high *ζ* suggests that viscous behavior dominates over elastic behavior, and indicates insufficient FSN network formation and a tendency for the printed resin to deform. For micro‐dispensing, an optimal *ζ* is typically between 0.1 and 0.4, which indicates a balance between elastic and viscous behavior for ML shape retention [36, 37]. The thixo‐PQD resins with 12 wt.% FSN had ζ = 0.112 and retained their printed shape (Figure 2a, Figure S5 and Table S1).

**TABLE 1:** Damping factor, shear viscosity, and shear‐thinning index of thixo‐PQD resin, thixo‐RQD resin, and thixo‐QD‐free resin.

FSN wt.%	Damping Factor ζ	Viscosity at 0.1 s^−1^ [mPa·s]	Shear‐thinning Index
Thixo‐PQD Resin			
0	207.67	2.27 * 10^3^	1.15
5	0.565	4.21 * 10^4^	8.72
10	0.144	6.02 * 10^5^	52.32
12	0.112	2.08 * 10^6^	77.01

Thixo‐GQD Resin			
0	190.30	2.09 * 10^3^	1.06
5	0.609	3.49 * 10^4^	7.98
10	0.161	5.19 * 10^5^	57.85
12	0.136	1.84 * 10^6^	80.73

Thixo‐RQD Resin			
0	200.14	2.12 * 10^3^	1.05
5	0.603	3.98 * 10^4^	8.04
10	0.156	5.64 * 10^5^	59.12
12	0.132	1.92 * 10^6^	81.97

Thixo‐QD‐Free Resin			
0	184.76	2.03 * 10^3^	1.09
5	0.646	3.01 * 10^4^	7.86
10	0.168	4.88 * 10^5^	56.39
12	0.137	1.41 * 10^6^	78.16

The shear‐thinning index is another critical factor for evaluating the feasibility of the micro‐dispensing process, expressed as follows in Equation (2):

(2) $$Shear-Thinning\;Index=\frac{\eta_{0.1}}{\eta_{50}}$$

where η_0.1_ and η_50_ are referred to viscosity values at shear rate 0.1 and 50 s^−1^, respectively. Increasing the FSN content from 0 to 12 wt.% significantly increased shear‐thinning index from 1.15 to 77.01, which means that viscosity decreased steeply as shear rate increased. This shear‐thinning behavior facilitates extrusion of the thixo‐PQD resin through the nozzle during the micro‐dispensing process [29, 38]. Stable extrusion requires a shear‐thinning index over 10; this criterion was achieved at FSN content > 5 wt.%, so the thixo‐PQD resin was suitable for the micro‐dispensing process (Figure 2b and Table 1).

During the practical micro‐dispensing process, shear force changes drastically before and after the extrusion step. In the three‐interval thixotropic test (3ITT) of PQD/siloxane resin and thixo‐PQD resin, the storage modulus of thixo‐PQD resin quickly drops into 0.90% of the initial value (72210 Pa at shear stress 9.5 Pa, 648 Pa at shear stress 2000 Pa; Figure S6). When the shear force is applied, *G*″ becomes larger than *G*'; this change indicates that resin flows easily when pneumatic pressure is applied to the thixo‐PQD resin. After the resin is dispensed, its *G*' recovers quickly; this trait provides shape fidelity to the ML thixo‐PQD array.

We further synthesized green‐emitting thixo‐GQD resin (by using green‐emitting Cd‐based QDs), red‐emitting thixo‐RQD resin (by using red‐emitting Cd‐based QDs), and non‐emitting thixo‐QD‐free resin (not containing luminescent QDs) to achieve a white‐emitting color conversion layer. Similar to thixo‐PQD resin, synthesized thixo‐GQD resin, thixo‐RQD resin, and thixo‐QD‐free resin had low ζ (thixo‐GQD resin: 0.136, thixo‐RQD resin: 0.132, and thixo‐QD‐free resin: 0.137) and high shear‐thinning index (thixo‐GQD resin: 80.73, thixo‐RQD resin: 81.97, and thixo‐QD‐free resin: 78.16) for uniform micro‐dispensing printing (Figures S7–S9 and Table S1).

To investigate the scattering effect of thixo‐PQD film, a flat thixo‐PQD film with 240 µm thickness was fabricated to compare total transmittance and direct transmittance (Figure 2c). Flat thixo‐PQD films showed significantly lower direct transmittance (18.1%) than total transmittance (62.3%), yielding high haze *H* = 70.9% (Table S2). The high *H* of the thixo‐PQD film yielded higher PLQY than did the PQD/siloxane film as the FSN content increased (thixo‐PQD film: 78.1%, PQD/siloxane film: 75.2%; Figure 2d). Moreover, the average PL lifetime of thixo‐PQD film was increased (thixo‐PQD film: 79.2 ns, PQD/siloxane film: 58.3 ns; Figure S10 and Table S3) from time‐correlated single‐photon‐counting (TCSPC) analysis of PQD/siloxane film and thixo‐PQD film. These results indicate the FSN network provides a significant scattering effect and higher optical path length [39]. Thermogravimetric analysis (TGA) showed an increase in 5 wt.% thermal decomposition temperature T_d5wt%_ to 346°C in thixo‐PQD film, from 281°C in PQD/siloxane film (Figure S11).

To support the scattering effect of the FSNs, high *H* was obtained in flat thixo‐GQD film (70.1%), flat thixo‐RQD film (75.8%), and flat thixo‐QD‐free film (36.9%) (Figures S12–S14 and Table S2). Furthermore, in all PQD/QD films that incorporated FSN, the PLQY increased as FSN content increased (Figures S15 and S16). These results indicate that the FSN material serves as an efficient scattering network that increases optical path length and thereby increases photoluminescence efficiency.

### 2.3 Color‐Conversion Properties of ML Thixo‐PQD Array

**FIGURE 3:**
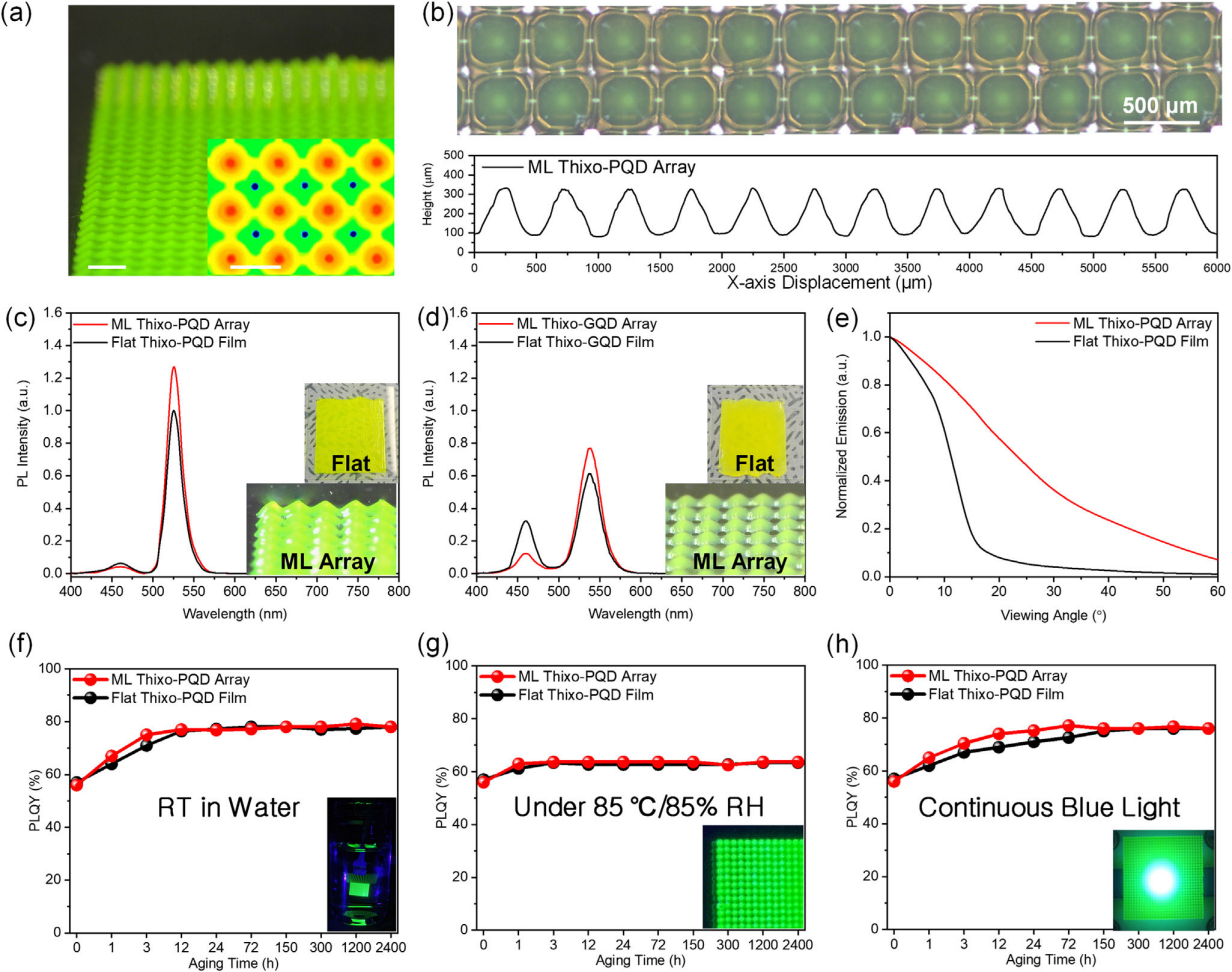
(a) Image and colored mapping of ML thixo‐PQD array (Scale bar: 500 µm). (b) 3D laser microscopic image and height profile of ML thixo‐PQD array (Scale bar: 500 µm). (c) PL spectra and inset images of ML thixo‐PQD array (red) and flat thixo‐PQD film (black). (d) PL spectra and inset images of ML thixo‐GQD array (red) and flat thixo‐GQD film (black). (e). Angular emission profile of ML thixo‐PQD array and flat thixo‐PQD film (black). (f–h) Stability tests of ML thixo‐PQD array (red) and flat thixo‐PQD film (black) under (f) RT in water, (g) 85°C/85% RH chamber, and (h) continuous blue light irradiation (current bias: 5 mA, luminous flux: 99 mlm, irradiance: 372 mW cm^−2^).

After repetitive micro‐dispensing cycles of gap‐adjustment and extrusion steps, thixo‐PQD resin was uniformly printed to form an ML thixo‐PQD array (Figure 3a). It exhibited a pixel pitch of 500.00 ± 0.31 µm (pixel density: 51 ppi), a maximum ML height of 320.08 ± 1.12 µm, and an overlapping height of 90.06 ± 0.66 µm between adjacent pixels (Figure 3b and Figure S17). From these geometrical parameters, the area‐averaged thickness of the ML thixo‐PQD array was calculated to be ∼ 190 µm. For comparison of color‐conversion characteristics, a flat thixo‐PQD film with 240 µm thickness was prepared by bar‐coating, yielding a higher thickness than that of the ML thixo‐PQD array.

Blue leakage (%) was defined as the ratio of the blue emission intensity (I_BL_) to the maximum blue excitation intensity (I_EX_), as expressed in Equation (3), and the relative green emission intensity was quantified according to Equation (4).

(3) $$Blue\;Leakage\;\left(\%\right)=I_{BL}\;/I_{EX}$$

(4) $$Relative\;Green\;Emission\;\left(\%\right)=I_{GR}\;/I_{EX}$$

The ML thixo‐PQD array exhibited reduced blue leakage (ML thixo‐PQD array: 1.67%, flat thixo‐PQD film: 2.92%) and 26% enhanced green emission compared with the flat film (Figure 3c and Table 2).

**TABLE 2:** Blue leakage and green/red emission ratio of flat thixo‐PQD film, flat thixo‐GQD film, flat thixo‐RQD film, ML thixo‐PQD array, ML thixo‐GQD array, and ML thixo‐RQD array.

Quantity	Flat Thixo‐PQD	Flat Thixo‐GQD	Flat Thixo‐RQD
Blue Leakage [%]	2.92	15.83	12.18
Green/red emission ratio [%]	37.76	23.13	29.48

Quantity	ML Thixo‐PQD	ML Thixo‐GQD	ML Thixo‐RQD
Blue Leakage [%]	1.67	5.92	3.25
Green/red emission ratio [%]	47.92	29.04	35.01

To further evaluate the effect of 3D architecture on color conversion, three ML arrays (ML thixo‐GQD array, ML thixo‐RQD array, and ML thixo‐QD‐free array) were fabricated using thixo‐GQD, thixo‐RQD, and thixo‐QD‐free resins, respectively. The average ML heights were 298 µm (ML thixo‐GQD array), 310 µm (ML thixo‐RQD array), and 302 µm (ML thixo‐QD‐free array) (Figures S18–S20). Both ML thixo‐GQD and ML thixo‐RQD arrays achieved substantially higher color‐conversion performance than their corresponding flat films (flat thixo‐GQD films and flat thixo‐RQD films) (Figure 3d, Figure S21 and Table S2); this result confirms that control of the optical path length and light confinement in the ML arrays increases color‐conversion efficiency. Moreover, the ML thixo‐PQD array showed even higher green emission intensity than the ML thixo‐GQD arrays, because of the intrinsically higher PL efficiency of the PQDs.

The viewing angle was 12° for the flat thixo‐PQD film, and 25° for the ML thixo‐PQD array (Figure 3e). Consequently, the ML thixo‐PQD array demonstrated directional behavior in PL emission with respect to the incident excitation angle (Figure S22). The PL intensity was maximized when the excitation light was incident below the ML structure, reflecting the geometric focusing effect. This improvement in angular luminance distribution and wider viewing angle is consistent with the behavior observed in the ML thixo‐GQD array (Figure S23). The increased viewing angle and PL emission are attributed to the 3D ML architecture, which refracts light in multiple directions and thereby increases color‐conversion efficiency. Collectively, the ML geometry reduces residual blue leakage, increases converted output, and mitigates angle‐dependent PL emission. These attributes make the MLs suitable for color‐converted micro‐LED applications that require high efficiency and wide‐angle consistency.

The environmental stability of the ML thixo‐PQD array is critical for ensuring long‐term operation of micro‐LED devices. Under storage at room temperature (RT) in ambient air or in water, the PLQY of the ML thixo‐PQD array increased from 57% to 78% within 3 h (Figure 3f and Figure S24). The PLQY of the flat thixo‐PQD films increased by the same amount, but required 12 h to reach 78%.

Similar trends were observed in harsh chemical environments: after immersion for 2400 h (100 d), PLQYs of 77% and 75% were maintained in 0.1 m HCl and 0.1 m NaOH, respectively, and 65% and 63% were retained in ethanol and isopropanol, respectively (Figure S25). We attribute this PLQY increase to defect passivation at the PQD surface, which proceeds more rapidly in water than in air, and in the ML thixo‐PQD array, which is thinner and has higher surface area than does a flat thixo‐PQD film [30, 31]. This increase represents a remarkable improvement over conventional PQDs, which typically degrade rapidly in humid or aqueous environments.

Environmental stability of the ML thixo‐PQD array when subjected to heat and light irradiation was also evaluated. Under 85°C/85% RH, the PLQY of both the ML thixo‐PQD array and flat thixo‐PQD film increased to 65% (Figure 3g,h). Under continuous blue‐light irradiation at a current bias of 5 mA (luminous flux: 99 mlm, irradiance: 372 mW cm^−2^), the PLQY did not decrease for 2400 h (Figure 3h). These PLQY increasing of ML thixo‐PQD arrays are supported by TCSPC analysis, which showed increased PL lifetime after stability testing, indicating reduced defect‐assisted recombination at the PQD surface (Figure S26 and Table S4). These findings indicate that the internal siloxane hybrid network in the ML thixo‐PQD array effectively protects the PQDs from the environment, and thus preserves their optical properties. In addition, the changes in optical transmittance of flat thixo‐PQD, flat thixo‐GQD, flat thixo‐RQD, and flat thixo‐QD‐free films after aging at high temperature were all < 5% (Figure S27); this result confirms that the optical properties of the siloxane/FSN matrix did not change. Collectively, these results demonstrate that all three thixotropic FSN‐siloxane resins provide highly stable color‐conversion layers under a wide range of environmental stresses.

### 2.4 White‐emitting ML CCL and Color‐Converted Micro‐LED

**FIGURE 4:**
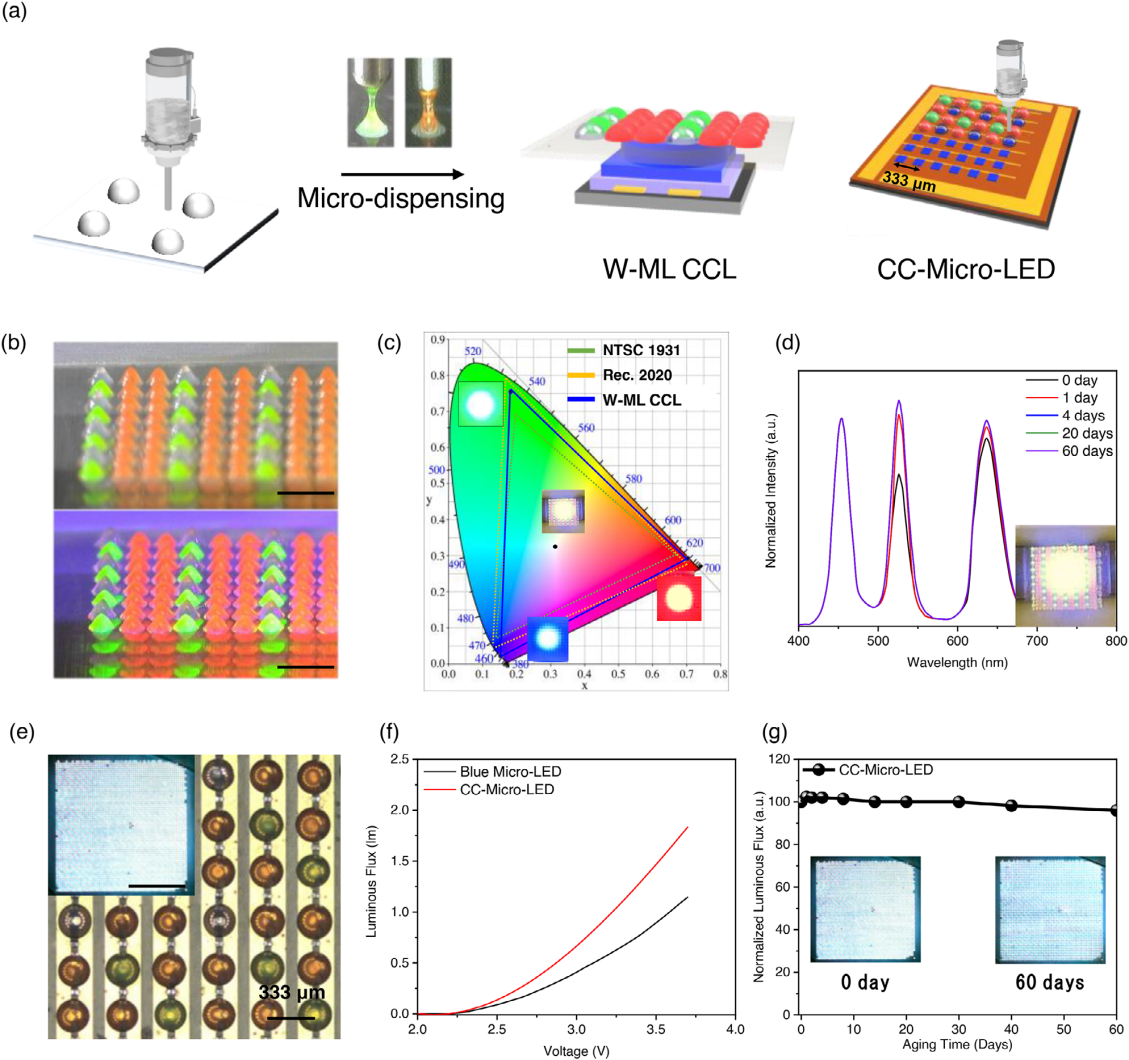
(a) Schematic illustration of white‐emitting ML CCL array (W‐ML CCL) and color‐converted micro‐LED (CC‐micro‐LED). (b) Image of W‐ML CCL. (c) PL spectra of W‐ML CCL during stability test under continuous blue light irradiation (current bias: 5 mA). (d) CIE 1931 color coordinate of W‐ML CCL. Insets: images of ML thixo‐PQD array, ML thixo‐RQD array, and ML thixo‐QD‐free array. (e) Microscopic image of CC‐micro‐LED (Scale bar: 333 µm). Inset: CC‐micro‐LED with driving current (Scale bar: 1 cm). (f) Luminous flux‐voltage curve of CC‐micro‐LED (red) and blue micro‐LED backplane (black). (g) Relative luminous flux of CC‐micro‐LED under 85°C/85% RH chamber.

We further realized a white‐emitting ML CCL (W‐ML CCL) by sequentially printing blue (thixo‐QD‐free resin), green (thixo‐PQD resin), and red (thixo‐RQD resin) pixels (Figure 4a,b). With the same micro‐dispensing parameters as the ML thixo‐PQD array, the W‐ML CCL had an average ML height of 290 µm with a pixel pitch of 500 µm (Figure S28). The blue, green, and red pixels emitted at CIE 1931 color coordinates of (0.153, 0.023), (0.181, 0.757), and (0.693, 0.297), respectively. The combined W‐ML CCL showed natural white emission with CIE coordinates (0.312, 0.336), and achieved a wide color gamut that covers 122.8% of the NTSC 1931 standard and 91.7% of the Rec. 2020 standard (Figure 4c). The W‐ML CCL also maintained their PLQY and distinct PL emission intensity under continuous blue‐light irradiation (Figure 4d and Figure S29); this result confirms that the multi‐color ML geometry does not compromise the stability of the underlying thixotropic resin system.

To validate the micro‐dispensing approach on devices, a full‐color CC‐micro‐LED array was demonstrated (Figure 4e). On a blue‐emitting micro‐LED substrate, the blue, green, and red pixels were sequentially and uniformly printed with an average pixel width of 300.14 ± 1.67 µm. The average pixel heights were 135.84 ± 0.88 µm for blue pixels, 140.04 ± 0.46 µm for green pixels, and 136.11 ± 0.83 µm for red pixels, confirming the uniform formation of an RGB microlens‐type color‐conversion array (Figure S30 and S31, Videos S1–S3). The center‐to‐center distance between adjacent pixels was 333 µm, corresponding to a pixel density of 76 ppi. The resulting CC‐micro‐LED achieved 98.22% pixel yield (compared to 98.29% for the bare blue‐emitting micro‐LED); this result indicates that the micro‐dispensing process did not damage the micro‐LED backplane. The uniformly patterned pixels had 59.2% greater luminous flux relative to the bare blue‐emitting micro‐LED device, and electroluminescence spectra confirmed efficient color‐conversion to white emission (Figure 4f and Figure S32). Furthermore, the CC‐micro‐LED retained its luminous flux after storage in an 85°C/85% RH chamber for > 60 d; this result emphasizes the exceptional reliability of the full‐color micro‐LED due to the environmentally stable red, green, and blue pixels that incorporate siloxane hybrid (Figure 4g). As a proof‐of‐concept for finer patterning, ML thixo‐PQD arrays with 48 µm pixel width and 70 µm pitch (363 ppi) were achieved (Figure S33). Overall, the W‐ML CCLs and the realized PQD CC‐micro‐LEDs demonstrate excellent optical characteristics and strong potential for use in efficient, reliable color‐converted micro‐LED displays.

To summarize, this study focuses on microlens‐type arrays by micro‐dispensing; the developed thixo‐PQD resin can be further extended toward high‐resolution color‐converted micro‐LED displays. The resin combines high PQD stability, chemical compatibility between PQD surface ligands and the siloxane hybrid matrix, and rheological shape retention, which are essential for forming stable color‐conversion pixels. To improve color‐conversion efficiency in smaller pixels, increasing PQD content and intrinsic emission efficiency while maintaining matrix passivation will be crucial, because a reduced pixel volume requires higher conversion characteristics per unit volume [40, 41]. In parallel, narrower nozzles, finely controlled dispensing heads, and narrower‐pitch micro‐LED backplanes should enable more uniform high‐resolution pixel formation [13]. Therefore, this printable and stable thixo‐PQD resin provides a promising material platform for future micropatterned advanced displays.

## 3 Conclusion

This paper has presented a method to achieve full‐color micro‐LED displays with color‐conversion materials by utilizing the micro‐dispensing process. The thixo‐PQD resin combines MAPbBr_3_ PQDs, a highly condensed siloxane hybrid matrix, and a percolated fumed‐silica nanoparticle (FSN) network, providing high PLQY, strong internal light scattering, and non‐Newtonian rheology suitable for precise micro‐dispensing. Micro‐dispensed ML thixo‐PQD arrays exploit both the FSN‐induced scattering and the dome‐shaped geometry to suppress blue leakage, increase color‐conversion efficiency, and improve viewing characteristics compared with compositionally identical flat films. Moreover, the fabricated ML thixo‐PQD array exhibited long‐term stability under ambient, high‐temperature, high‐humidity, and blue‐light irradiation. Extending this strategy to multicolor operation, we fabricated a W‐ML CCL that achieved a wide color gamut that covered 122.8% of the NTSC 1931 standard and 91.7% of the Rec. 2020 standard. Finally, the full‐color CC‐micro‐LED was realized by directly printing the thixotropic resins onto blue micro‐LED backplanes, achieving a 59.2% increase in luminous flux and high reliability under 85°C/85% RH. These results demonstrate that micro‐dispensing of ML architectures that incorporate thixo‐PQD resin offers a promising approach to fabrication of efficient and reliable micro‐LED displays.

## 4 Experimental Methods

### 4.1 Materials

Methylammonium bromide (MABr) was purchased from Greatcellsolarmaterials, Australia. Lead bromide (PbBr_2_) was purchased from TCI, Japan. MPTS were purchased from ShinEtsu, Japan. DPSD was purchased from Gelest, U.S.A. N, N‐dimethylformamide, toluene, 1‐butanol, hexane, oleic acid, and barium hydroxide monohydrate (Ba(OH)_2_·H_2_O) were purchased from Sigma–Aldrich, U.S.A. CdS/CdSe QDs (Red QDs and Green QDs, passivated by oleic acid and oleylamine) were purchased from FineLab, Republic of Korea. 1‐[4‐(Phenylthio)phenyl]‐1,2‐octanedione 2‐(O‐benzoyloxime) (OXE‐01) was purchased from BASF, Germany. Fumed silica nanoparticles (FSN) were purchased from Evonik, Germany.

### 4.2 Synthesis of Green‐Emitting Thixo‐PQD Resin

Silane‐passivated green‐emitting MAPbBr_3_ PQDs were synthesized using a modified ligand‐assisted reprecipitation method that was reported previously [30]. Green‐emitting PQD/siloxane resin was synthesized by the in situ sol–gel process that used MPTS and DPSD (1:1 molar ratio) in the presence of PQDs. The silane precursors (MPTS and DPSD) and colloidal PQD solution (1.2 wt.% of total resin mass) were mixed using a magnetic stirrer in a two‐neck flask with a base catalyst to promote sol–gel condensation that formed a siloxane network. Then the temperature was elevated to 80°C with N_2_ purging. After 4 h, FSN particles were added to 5, 10, and 12 wt.% of thixo‐PQD resin amounts. To solidify it, OXE01 with 2 wt.% of the obtained resin was added to initiate a UV‐induced free‐radical addition reaction.

For comparison of color‐conversion characteristics and fabrication of white‐emitting CCLs, thixo‐GQD resin, thixo‐RQD resin, and thixo‐QD‐free resin were prepared using the same in situ sol–gel process. The thixo‐GQD resin contained 1.2 wt.% green QDs with respect to the total resin mass, whereas the thixo‐RQD resin contained 1.2 wt.% red QDs with respect to the total resin mass. The thixo‐QD‐free resin was prepared without adding luminescent QDs, while maintaining the same siloxane resin composition, FSN content, and OXE01 photoinitiator content as those used for the thixo‐GQD resin or thixo‐RQD resin.

### 4.3 Fabrication of ML Thixo‐PQD Array and Flat PQD Films

The ML thixo‐PQD array was fabricated using a micro‐dispensing method, using a Shot Mini 200S dispenser (Musashi Engineering, Inc., Japan) with a 25G needle (inner diameter: 250 µm) [28, 29]. The dispenser head was mounted on a motorized stage equipped with a Dino‐Lite 1.3MP optical microscope and actuated by pneumatic pressure (0.5 MPa). The printing program was set to execute a repetitive sequence in which the dispensing cylinder containing the thixo‐PQD resin moved downward to the substrate, extruded resin for 2.0 s, then retracted at 1.5 mm/s. The center‐to‐center spacing between adjacent domes was 500 µm. After the ML pattern had been formed, it was exposed to UV (λ = 365 nm) to yield a solidified ML thixo‐PQD array. For comparison with the ML thixo‐PQD films, flat thixo‐PQD films were also fabricated. The thixo‐PQD resin was coated using a bar‐coating method, then UV‐cured for 5 min under N_2_ purging to yield a 240 µm thickness flat films.

The W‐ML CCLs were fabricated by sequential micro‐dispensing of thixo‐QD‐free resin, thixo‐PQD resin (1.2 wt.% PQD), and thixo‐RQD resin (1.0 wt.% RQD), with the same micro‐dispensing parameters as for the ML thixo‐PQD array. The red‐emitting thixo‐RQD resin was also deliberately formulated with 1.0 wt.% RQDs to control the red emission contribution and match the green component in the W‐ML CCL. In each unit cell, one QD‐free (transmitting blue backlight) pixel, one green PQD pixel, and four RQD pixels were sequentially printed to form a six‐pixel W‐ML CCL. The UV‐curing conditions were identical to those described above.

### 4.4 Fabrication of CC‐Micro‐LED

The CC‐micro‐LED was fabricated using different micro‐dispensing parameters as follows: 30G needle (inner diameter 150 µm), dispensing time 2.5 s, pneumatic pressure 0.6 MPa, and retraction speed 1.5 mm/s. The center‐to‐center spacing between domes was 333 µm. For each unit cell, one QD‐free (transmitting blue emission from micro‐LED) pixel, two green‐emitting pixels (thixo‐PQD resin) were sequentially printed (Videos S1–S3), forming nine ML pixels per CC‐micro‐LED unit.

### 4.5 Stability Tests

Stability tests were conducted to observe the consistent optical properties of ML thixo‐PQD array. To test stability against air, water, and heat, ML thixo‐PQD arrays were placed in ambient air at RT or in water at RT, or in a temperature/humidity chamber with 85°C/85% RH. To test chemical stability, a ML thixo‐PQD array was soaked in 0.1 m HCl, 0.1 m NaOH, ethylene glycol, ethanol, or isopropanol. To test photostability, the ML thixo‐PQD array was directly placed on a blue LED chip in ambient air. Stability tests of W‐ML CCL were also evaluated by the same method. To test the reliability of the fabricated CC‐micro‐LED, it was placed in an 85°C/85% RH chamber, and opto‐electrical properties were measured at regular intervals.

### 4.6 Characterization

The rheological properties were analyzed using a rheometer (MCR 302; Anton Paar, Austria) that uses parallel plates with a diameter of 25.012 mm at a constant 25°C. FT‐IR spectra were obtained using an FTIR spectrometer (Nicolet Summit X; Thermo Fisher Scientific, U.S.A.). The ^29^Si‐NMR spectrum was collected using a DMX600 FT 600 MHz (Bruker Biospin, Australia). TEM images were obtained using a field‐emission electron microscope (JEM‐2100F HR; JEOL, Japan) on specimens obtained using a focused‐ion‐beam generator (Nova‐200; FEI Company, Netherlands). SAXS profiles were obtained by NANOPIX (RIGAKU, Japan). Powder XRD patterns were obtained using an X‐ray diffractometer (D/MAX‐2500, RIGAKU, Japan). Dispersive Raman PL spectrometry and 2D mapping images were collected using an ARAMIS (Horiba Jobin Yvon, France). 3D elemental mapping images of TOF‐SIMS were obtained using a time‐of‐flight secondary‐ion mass spectrometer (ION‐TOF; GmbH, Germany), with Bi^+^ (30 keV energy), O^2−^ (0.5 keV energy), and Cs^+^ (1 keV energy) ion beams. TGA was performed using a simultaneous thermogravimetric analyzer (STA200; Hitachi, Japan) at a ramp rate of 10°C·min^−1^ under N_2_ gas. PLQY was obtained using a photoluminescence quantum‐yield spectrometer (Quantaurus‐QY C11347 series; Hamamatsu Photonics, K.K., Japan) with a 150 W Xenon light source. The excitation wavelength for measuring PLQY was 460 nm. PL spectra and CIE color coordinates were recorded on a Diode Array Rapid Scan Analysis system (DARSA PRO 5100 PL; PSI Trading Co., Ltd., Korea). The absorption and transmittance spectra were measured using a UV–vis–IR spectrophotometer (SolidSpec‐3700; Shimadzu, Japan). PL decay curves were recorded using a Fluorescence Spectrometer (FL920; Edinburgh Instruments, England), with an excitation wavelength 405 nm and a slit width 5 nm. Height profiles and corresponding microscope images were obtained using a 3D laser microscope (VK‐X1050; Keyence, Japan). SEM images were obtained using a Schottky field‐emission scanning electron microscope (SU‐5000; Hitachi, Japan). The viewing angle was measured using a Conometer (CA‐2500A, Konica Minolta, Canada). PL spectra according to the incident excitation angle were measured by a light‐source‐extendable visible‐to‐NIR spectrofluorometer NFEC‐2024‐12‐302228 (Fluoromax Plus; Horiba, Japan). A blue LED (EBT801‐S‐801; Seoul Semiconductor, Korea) was used as the light source, with a film placed on top during the measurement. Driving current, voltage, and luminous flux of the CC‐micro‐LED were measured using an LED wafer‐level measurement system (OPI‐160; Withlight, Korea).

## Conflicts of Interest

The authors declare no conflicts of interest.

## Supporting information




**Supporting File 1**: smll74347‐sup‐0001‐SuppMat.docx.

**Supporting Video 2**: smll74347‐sup‐0002‐VideoS1.mp4.

**Supporting Video 3**: smll74347‐sup‐0003‐VideoS2.mp4.

**Supporting Video 4**: smll74347‐sup‐0004‐VideoS3.mp4.

## Data Availability

The data that support the findings of this study are available from the corresponding author upon reasonable request.
