# Supplementary material for: Printable and Stable Microlens‐Type Perovskite Quantum Dot Color‐Converting Resin Through Rheology Control for Efficient and Reliable Micropatterning for Full‐Color Displays
Source: Small. 2026 Jun 26;22(46):e74347. doi: 10.1002/smll.74347 (PMC13485040; doi:10.1002/smll.74347)
Supplement: Supplementary file 1 [file SMLL-22-e74347-s003.docx]

Supporting Information

**Printable and Stable Microlens-type Perovskite Quantum Dot Color-Converting Resin through Rheology Control for Efficient and Reliable Micropatterning for Full-Color Displays**

*Yongmin Shin^†^, Jiho Joo^†^, Byung Jo Um, Jung Ho Shin, Chanmi Lee, Somin Park, Junho Jang^*^, Byeong-Soo Bae^*^*

Y. Shin, B. J. Um, S. Park, B.-S. Bae

Wearable Platform Materials Technology Center (WMC), Department of Materials Science and Engineering, Korea Advanced Institute of Science and Technology (KAIST), Daehak-ro 291, Yuseong-gu, Daejeon 34141, Republic of Korea.

E-mail: bsbae@kaist.ac.kr

J. Joo, J. H. Shin, C. Lee

Superintelligence Creative Research Laboratory, Electronics and Telecommunications Research Institute (ETRI), Gajeong-ro 218, Yuseong-gu, Daejeon 34129, Republic of Korea

J. Jang

Insulation Materials Research Center, Electrical Materials Research Division, Korea Electrotechnology Research Institute (KERI), 12, Jeongiui-gil, Seongsan-gu, Changwon-si, Gyeongsangnam-do 51543, Republic of Korea

E-mail: jhjang0427@keri.re.kr

Keywords: perovskite quantum dot, siloxane hybrid, thixotropic resin, stability, color-conversion, micro-LED display

^†^Y. Shin and J. Joo contributed equally to this work.

**Figure S1.** ^29^Si-NMR spectrum of thixo-PQD resin.

**Figure S2.** XRD patterns of MAPbBr_3_ PQD (black), PQD/siloxane film (red), and thixo-PQD film (blue).

**Figure S3.** TEM images of thixo-PQD film of (a) FSN 5 wt%, (b) FSN 10 wt%, and (c) FSN 12 wt%.

**Figure S4.** Dispersive RAMAN/PL spectrum and mapping of thixo-PQD film.

**Figure S5.** Storage modulus (bold line) and loss modulus (broken line) of thixo-PQD resin and PQD/siloxane resin.

**Figure S6.** (a) 3ITT test of thixo-PQD resin (Bold line: storage modulus, broken line: loss modulus). (b) Shear stress curve during 3ITT test.

**Figure S7.** (a) Flow curve of thixo-GQD resin. (b) Storage modulus (bold line) and loss modulus (broken line) of thixo-GQD resin.

**Figure S8.** (a) Flow curve of thixo-RQD resin. (b) Storage modulus (bold line) and loss modulus (broken line) of thixo-RQD resin.

**Figure S9.** (a) Flow curve of thixo-QD-free resin. (b) Storage modulus (bold line) and loss modulus (broken line) of thixo-QD-free resin.

**Figure S10.** PL decay curve of thixo-PQD resin by FSN content.

**Figure S11.** TGA profile of thixo-PQD film by FSN content.

**Figure S12.** (a) Total transmittance (black) and direct transmittance (red) of flat thixo-GQD film. (b) Total transmittance (black) and direct transmittance (red) of flat thixo-GQD film.

**Figure S13.** (a) Total transmittance (black) and direct transmittance (red) of flat thixo-RQD film. (b) Total transmittance (black) and direct transmittance (red) of flat thixo-RQD film.

**Figure S14.** (a) Total transmittance (black) and direct transmittance (red) of flat thixo-QD-free film. (b) Total transmittance (black) and direct transmittance (red) of flat thixo-QD-free film.

**Figure S15.** PL spectra of red-emitting RQD solution (black), RQD/siloxane resin (red), and thixo-RQD resin (blue).

**Figure S16.** PLQY of red thixo-RQD film.

**Figure S17.** (a) Pixel width distribution measured from 400 pixels at ML thixo-PQD array. Inset: optical image of the analyzed 400 pixels. (b) Pixel height distribution of the ML thixo-PQD array; the microlens pixel height is indicated in the inset image. (c) Overlapping height distribution of the ML thixo-PQD array; the overlap height between adjacent pixels is indicated in the inset image.

**Figure S18.** (a) 3D laser microscopic image (scale bar: 500 μm) and height profile of ML thixo-GQD array. (b) Dino-Lite 1.3 MP microscopic image (mounted on the dispenser system) of ML thixo-GQD array (scale bar: 1000 μm). (c) Height color mapping of ML thixo-GQD array (scale bar: 500 μm). Color scale on the right indicates heights from 0 μm (dark blue) to the 300 μm (red).

**Figure S19.** (a) 3D laser microscopic image (scale bar: 500 μm) and height profile of ML thixo-RQD array. (b) Dino-Lite 1.3 MP microscopic image (mounted on the dispenser system) of ML thixo-RQD array (scale bar: 1000 μm). (c) Height color mapping of ML thixo-RQD array (scale bar: 500 μm). Color scale on the right indicates heights from 0 μm (dark blue) to the 300 μm (red).

**Figure S20.** (a) 3D laser microscopic image (scale bar: 500 μm) and height profile of ML thixo-QD-free array. (b) Dino-Lite 1.3 MP microscopic image (mounted on the dispenser system) of ML thixo-QD-free array (scale bar: 1000 μm). (c) Height color mapping of ML thixo-QD-free array (scale bar: 500 μm). Color scale on the right indicates heights from 0 μm (dark blue) to the 300 μm (red).

**Figure S21.** (a) PL spectra of flat thixo-PQD film (black), ML thixo-PQD array (red), and blue LED (blue). (b) PL spectra of flat thixo-GQD film (black), ML thixo-GQD array (red), and blue LED (blue). (c) PL spectra of flat thixo-RQD film (black), ML thixo-RQD array (red), and blue LED (blue).

**Figure S22.** (a) Normalized PL intensity of ML thixo-PQD array and flat thixo-PQD film according to incident excitation angle. (b) PL spectra of ML thixo-PQD array and (c) PL spectra of flat thixo-PQD film according to incident excitation angle. (d) Normalized PL intensity of ML thixo-PQD array and flat thixo-PQD film according to incident excitation angle, with flipped measurement. (e) PL spectra of ML thixo-PQD array and (f) PL spectra of flat thixo-PQD film according to incident excitation angle.

**Figure S23.** Angular emission profile of ML thixo-GQD array (red) and flat thixo-GQD film (black).

**Figure S24.** Stability of ML thixo-PQD array (red) and flat thixo-PQD film (black) under RT in ambient air.

**Figure S25.** (a) Stability of ML thixo-PQD array soaking under 0.1 M HCl (red) and 0.1 M NaOH aqueous solution (black). (b) Stability of ML thixo-PQD array soaking under isopropanol (red) and ethanol (black).

**Figure S26.** PL decay curve of ML thixo-PQD array. Immediately after UV-curing (As-cured, black), after stability test under RT in water for 300 h (red), after stability test under 85 ℃/85% RH for 300 h (blue), and after stability test under blue light irradiation for 300 h (green).

**Figure S27.** Transmittance before and after stability test of (a) flat thixo-PQD film, (b) flat thixo-GQD film, (c) flat thixo-RQD film, and (d) flat thixo-QD-free film. Flat thixo-PQD films were stored at 60°C/5% RH chamber for 30 days. Flat thixo-GQD and flat thixo-RQD films were stored at 80°C/5% RH chamber for 30 days. Flat thixo-QD-free films were stored at 120°C/5% RH chamber for 30 days.

**Figure S28.** (a) 3D laser microscopic image of W-ML CCL (scale bar: 500 μm). (b-c) Height profile 1 and 2 of W-ML CCL. Profile 1 contains green pixels and red pixels and profile 2 contains blue pixels and red pixels.

**Figure S29.** PLQY of W-ML CCL under continuous blue-light irradiation (current bias: 5 mA).

**Figure S30.** Sequential micro-dispensing process for fabricating the CC-micro-LED. (a) Blue micro-LED backplane, (b) dispensing of blue pixels, (c) dispensing of green pixels, and (d) dispensing of red pixels (scale bar: 1000 μm).

**Figure S31.** (a) Pixel width distribution measured from 540 pixels at CC-micro-LED. Inset: optical image of the analyzed 540 pixels, where one unit cell consists of two green pixels, six red pixels, and one blue pixel. (b) Pixel height distribution measured from 540 pixels at CC-micro-LED.

**Figure S32.** Electroluminescence spectra of CC-micro-LED (red) and blue micro-LED (black).

**Figure S33.** (a) 3D laser microscopic image of ML thixo-PQD array of 70 µm pixel width (scale bar: 70 µm). (b) Micro-dispensing using tapered needle (inner diameter: 30 µm). (c) SEM image of ML thixo-PQD array (scale bar: 70 µm). (d) 3D laser microscopic image of ML thixo-PQD array of 48 µm pixel width (scale bar: 70 µm) and its (e) SEM image (scale bar: 50 µm).

**Table S1.** PL emission properties (PLQY, FWHM, emission wavelength) of PQD, thixo-PQD resin, and thixo-PQD film.

| **Quantity** | **PQD Solution** | **Thixo-PQD resin** | **Thixo-PQD Film** |
| --- | --- | --- | --- |
| PLQY [%] | 80 | 80 | 77 |
| FWHM [nm] | 25 | 25 | 25 |
| Emission Peak [nm] | 523 | 523 | 525 |

**Table S2.** Total transmittance (Total T), direct transmittance (Direct T), and haze of flat thixo-PQD film, flat thixo-GQD film, flat thixo-RQD film, and flat thixo-QD-free film.

| **Quantity** | **Thixo-PQD** | **Thixo-GQD** | **Thixo-RQD** | **Thixo-QD-Free** |
| --- | --- | --- | --- | --- |
| Total T [%] | 62.3 | 72.1 | 77.7 | 92.5 |
| Direct T [%] | 18.1 | 21.4 | 18.8 | 58.4 |
| Haze [%] | 70.9 | 70.3 | 75.8 | 36.9 |

Transmittance was measured to each emission wavelength of given films. (Flat thixo-PQD film: 525 nm, flat thixo-GQD film: 530 nm, flat thixo-RQD film: 630 nm, and flat thixo-QD-free film: 460 nm). Haze *(H)* was quantified as below equation:

$H \left( \% \right)=(1- \frac{Direct T}{Total T}) X 100$	(1)

**Table S3.** PL lifetime of thixo-PQD film by FSN content.

| **FSN wt%** | **Average Lifetime [ns]** |
| --- | --- |
| 0 | 58.12 |
| 5 | 65.35 |
| 10 | 72.08 |
| 12 | 79.24 |

**Table S4.** PL lifetime of ML thixo-PQD array before and after stability test.

| **Stability Tests** | **Average Lifetime [ns]** |
| --- | --- |
| After UV-Curing (Before Stability Test) | 71.42 |
| RT in Water | 78.62 |
| 85 ℃/85% RH | 77.31 |
| Blue Light Irradiation | 79.24 |
